# Ticagrelor and preconditioning in patients with stable coronary artery disease (TAPER-S): a randomized pilot clinical trial

**DOI:** 10.1186/s13063-020-4116-7

**Published:** 2020-02-17

**Authors:** D. D’Amario, A. Restivo, A. M. Leone, R. Vergallo, S. Migliaro, F. Canonico, M. Galli, C. Trani, F. Burzotta, C. Aurigemma, G. Niccoli, A. Buffon, R. A. Montone, A. Flex, F. Franceschi, G. Tinelli, U. Limbruno, F. Francese, I. Ceccarelli, J. A. Borovac, I. Porto, F. Crea

**Affiliations:** 1 Fondazione Policlinico A. Gemelli IRCCS – Università Cattolica del Sacro Cuore, Largo Agostino Gemelli 8, 00168 Rome, Italy; 2 Dipartimento Cardio neuro vascolare, Azienda USL Toscana Sud-est, Ospedale di Grosseto, Grosseto, Italy; 3 Department of Pathophysiology, University of Split School of Medicine (USSM) and University Hospital Center Split (UHC Split), Split, Croatia; 4 Ospedale Policlinico San Martino IRCCS, Università degli Studi di Genova, Genoa, Italy

**Keywords:** Angina, Ischemic preconditioning, Ticagrelor, Clopidogrel, Adenosine, Fractional flow reserve, Intracoronary electrocardiography, Microvascular function, Microvascular dysfunction, Microvascular resistance

## Abstract

**Background:**

Ticagrelor is a reversibly binding, direct-acting, oral, P_2_Y_12_ antagonist used for the prevention of atherothrombotic events in patients with coronary artery disease (CAD). Ticagrelor blocks adenosine reuptake through the inhibition of equilibrative nucleoside transporter 1 (ENT-1) on erythrocytes and platelets, thereby facilitating adenosine-induced physiological responses such as an increase in coronary blood flow velocity. Meanwhile, adenosine plays an important role in triggering ischemic preconditioning through the activation of the A1 receptor. Therefore, an increase in ticagrelor-enhanced adenosine bioavailability may confer beneficial effects through mechanisms related to preconditioning activation and improvement of coronary microvascular dysfunction.

**Methods:**

To determine whether ticagrelor can trigger ischemic preconditioning and influence microvascular function, we designed this prospective, open-label, pilot study that enrolled patients with stable multivessel CAD requiring staged, fractional flow reserve (FFR)-guided percutaneous coronary intervention (PCI). Participants will be randomized in 1:1 ratios either to ticagrelor (loading dose (LD) 180 mg, maintenance dose (MD) 90 mg bid) or to clopidogrel (LD 600 mg, MD 75 mg) from 3 to 1 days before the scheduled PCI. The PCI operators will be blinded to the randomization arm. The primary endpoint is the delta (difference) between ST segment elevations (in millimeters, mm) as assessed by intracoronary electrocardiogram (ECG) during the two-step sequential coronary balloon inflation in the culprit vessel. Secondary endpoints are 1) changes in coronary flow reserve (CFR), index of microvascular resistance (IMR), and FFR measured in the culprit vessel and reference vessel at the end of PCI, and 2) angina score during inflations. This study started in 2018 with the aim of enrolling 100 patients. Based on the rate of negative FFR up to 30% and a drop-out rate up to 10%, we expect to detect an absolute difference of 4 mm among the study arms in the mean change of ST elevation following repeated balloon inflations. All study procedures were reviewed and approved by the Ethical Committee of the Catholic University of Sacred Heart.

**Discussion:**

Ticagrelor might improve ischemia tolerance and microvascular function compared to clopidogrel, and these effects might translate to better long-term clinical outcomes.

**Trial registration:**

EudraCT No. 2016–004746-28. No. NCT02701140.

**Trial status:**

Information provided in this manuscript refers to the definitive version (n. 3.0) of the study protocol, dated 31 October 2017, and includes all protocol amendments. Recruitment started on 18 September 2018 and is currently ongoing. The enrollment is expected to be completed by the end of 2019.

**Trial sponsor:**

Fondazione Policlinico Universitario A. Gemelli – Roma, Polo di Scienze Cardiovascolari e Toraciche, Largo Agostino Gemelli 8, 00168 Rome, Italy.

## Background

### Rationale

Ticagrelor is a reversibly binding, direct-acting, oral P_2_Y_12_ antagonist used for the prevention of atherothrombotic events in patients with acute coronary syndrome (ACS) [1]. It does not belong to thienopyridines; it is a carbocyclic nucleoside, representing a “first-in-class” cyclopentyl-triazolo-pyrimidine. In two phase II studies, dyspnea was noted to occur as a side effect to ticagrelor in a dose-dependent fashion, and in the PLATO study, a 6% absolute excess of dyspnea was observed in ticagrelor-treated patients compared with patients treated with clopidogrel [1]. In the ONSET/OFFSET study, dyspnea was more commonly associated with ticagrelor therapy in comparison with clopidogrel and placebo in patients with stable coronary artery disease (38.6%, 9.3%, and 8.3%, respectively) but was not associated with any adverse change in cardiac or pulmonary function [2]. Similar results were later confirmed in the population of patients with ACS [3].

The mechanisms for this side effect are largely unknown, although early data indicate that ticagrelor blocks adenosine reuptake through inhibition of ENT-1 transporter by erythrocytes [4], and intravenous adenosine infusion can cause transient dyspnea in the absence of bronchoconstriction [5]. Another mechanism potentially increasing adenosine levels by ticagrelor is reflected in adenosine triphosphate (ATP) release from erythrocytes [6]. Moreover, the biochemical comparison between ticagrelor and adenosine suggests their molecular similarity. Adenosine is a well-known key endogenous molecule that regulates tissue functions by activating four G-protein–coupled adenosine receptors: A1, A2A, A2B, and A3. Adenosine accumulates in the extracellular space in response to metabolic stress and cell damage, and elevations of adenosine are commonly encountered in conditions such as ischemia, hypoxia, inflammation, and trauma. Adenosine confers protective effects on the cell due to its anti-inflammatory, cardioprotective, cerebroprotective, antisclerotic, and antifibrotic properties, as well as by platelet inhibition and vasodilation [7].

Because to these effects of ticagrelor on adenosine biochemistry, chronic adenosine overload induced by ticagrelor was hypothesized to possibly contribute to the vascular outcome benefit observed in the PLATO trial, in addition to the inhibitory effect it has shown on platelet activity via P_2_Y_12_ receptor inhibition. Very recently, ticagrelor has been shown to increase adenosine-induced physiological responses in healthy human subjects by shifting the dose-response curve for adenosine-induced coronary blood flow velocity (CBFV) to the left and increasing area under the curve of CBFV [8]. Moreover, among patients with non–ST-segment elevation ACS that were treated with percutaneous coronary intervention (PCI) and received a maintenance dose of ticagrelor, a greater CBFV was observed compared to patients that received prasugrel maintenance dose in response to increasing adenosine concentrations [9]. These effects seem consistent with pharmacologically observed blockade of adenosine reuptake, as one of the pleiotropic effects exerted by the ticagrelor. An increase in adenosine bioavailability, as potentiated by ticagrelor, could have beneficial effects by both increasing coronary perfusion and enhancing the function of the microcirculation.

#### Activation of preconditioning

Ischemic preconditioning, consisting in episodes of ischemia as short as 5 min, followed by reperfusion, has been showed to protect the heart from a subsequent longer coronary artery occlusion by markedly reducing the amount of necrosis. Adenosine plays a key role in triggering ischemic preconditioning. Indeed, the stimulation of A1 adenosine receptors triggers a complex pathway that includes the epsilon isoform of protein kinase C, the ATP-dependent potassium channels, and the mitochondrial permeability transition pores, as well as other cellular pathways, like a paradoxical protective release of oxygen radicals eventually making cardiomyocytes more resistant to ischemia [10, 11]. In humans, examples of preconditioning are the pre-infarction angina and the angina “warm-up” phenomenon. Preconditioning can be reproduced experimentally by repetitive and sequential balloon inflations in the culprit coronary artery during the PCI, subsequently producing less chest pain and decrease in ST-segment elevation [12, 13].

Pharmacological preconditioning can be induced by intravenous or intracoronary administration of adenosine or A1 agonists of adenosine [14]. In a recent study performed in rabbits, investigators observed cardioprotective effects of clopidogrel and cangrelor (the intravenous analogue of ticagrelor) that were not attributed to the platelet inhibition but rather to activation of the signal transduction pathways of pre- and postconditioning, involving the reperfusion injury salvage kinases (RISK), including Akt and extracellular-regulated kinase (ERK), as well as adenosine A2B receptors, mitochondrial KATP channels, and redox signaling [15]. This cardioprotective effect of cangrelor was confirmed in a primate animal model as well [16, 17].

#### Improvement of coronary microvascular dysfunction

Coronary microvascular dysfunction has been demonstrated to affect the prognosis of ACS patients. Of note, Furber et al. described that doppler flow velocity parameters in the infarct-related artery are of prognostic value for long-term cardiac events [18]. Additionally, Takahashi et al. found an impaired coronary flow reserve velocity (CFVR) in the infarct-related artery to be significantly associated with increased cardiac event rates at long-term follow-up [19]. Furthermore, a microvascular function has been demonstrated to be altered even in nonischemic regions at distance from the infarcted myocardial tissue [20], and van de Hoef et al. have recently shown that microvascular dysfunction determined in the reference vessel after PCI is associated with a significantly increased long-term cardiac mortality [21].

Microvascular dysfunction is likely to occur also in the setting of non-ST-elevation ACS. In a cohort of 83 non-ST-elevation myocardial infarction (NSTEMI) patients, microvascular obstruction, as assessed by an elevated index of microcirculatory resistance (IMR) that is correlated with microvascular obstruction, was detected by the magnetic resonance imaging and was an independent predictor of adverse cardiovascular outcomes [22]. Marzilli et al. found that in patients with unstable angina, episodes of transient myocardial ischemia at rest are associated with a brisk increase in coronary microvascular resistance and that this increase is prevented by the administration of antiplatelet drugs [23]. Equally important, microvascular dysfunction may also occur following successful PCI; that is, coronary flow reserve has been shown to be impaired in the vascular bed subtended by the treated artery and requires up to 3 months for this microvascular dysfunction to resolve [24]. Finally, coronary microvascular reactivity to adenosine predicted adverse outcomes in women evaluated for suspected ischemia as demonstrated in WISE study [25].

### Pharmacology

#### Mechanism of action (taken from the SmPC of the drug in study)

Ticagrelor, a member of the chemical class cyclopentyl triazolo pyrimidines (CPTP), is an oral, direct-acting, selective, and reversibly binding P_2_Y_12_ receptor antagonist that prevents adenosine diphosphate (ADP)-mediated P_2_Y_12_ dependent platelet activation and aggregation [26]. Ticagrelor does not prevent ADP binding but when bound to the P_2_Y_12_ receptor prevents ADP-induced signal transduction. Since platelets participate in the initiation and/or evolution of thrombotic complications of the atherosclerotic disease, inhibition of platelet function has been shown to reduce the risk of cardiovascular (CV) events such as death, myocardial infarction, or stroke [27].

Ticagrelor also increases local endogenous adenosine levels by inhibiting the equilibrated nucleoside transporter-1 (ENT-1). Ticagrelor has been documented to augment the following adenosine-induced effects in healthy subjects and in patients with ACS: vasodilation (measured by coronary blood flow increases in healthy volunteers and ACS patients), inhibition of platelet function (in human whole blood in vitro), and dyspnea [28, 29]. However, a link between the observed increases in adenosine and clinical outcomes (e.g., morbidity-mortality) has not been clearly elucidated [11, 30].

## Objectives

The main goal of this study is to assess the pleiotropic effects of ticagrelor that could represent possible mechanisms for its beneficial effects on cardiovascular mortality and adverse outcomes.

### Primary objective

Ticagrelor may trigger ischemic preconditioning as compared to clopidogrel in patients with stable multivessel CAD that are undergoing staged PCI.

### Secondary objectives

Ticagrelor may improve microvascular perfusion in the myocardium of patients with stable multivessel CAD that are undergoing staged PCI.

### Study endpoints

#### Primary endpoint

The primary endpoint is the comparison of ticagrelor and clopidogrel for the delta (difference in mm) of the ST-segment elevation, as assessed by the intracoronary ECG during two-step sequential coronary balloon inflation in the index vessel.

#### Secondary endpoints

Secondary endpoints include the following:

1. Comparison of ticagrelor and clopidogrel on CFR, IMR, and FFR measured in the index vessel at the end of the PCI
2. Comparison of ticagrelor and clopidogrel on angina score during coronary balloon inflation

## Materials/Methods

### TAPER-S trial design

**Fig. 1:**
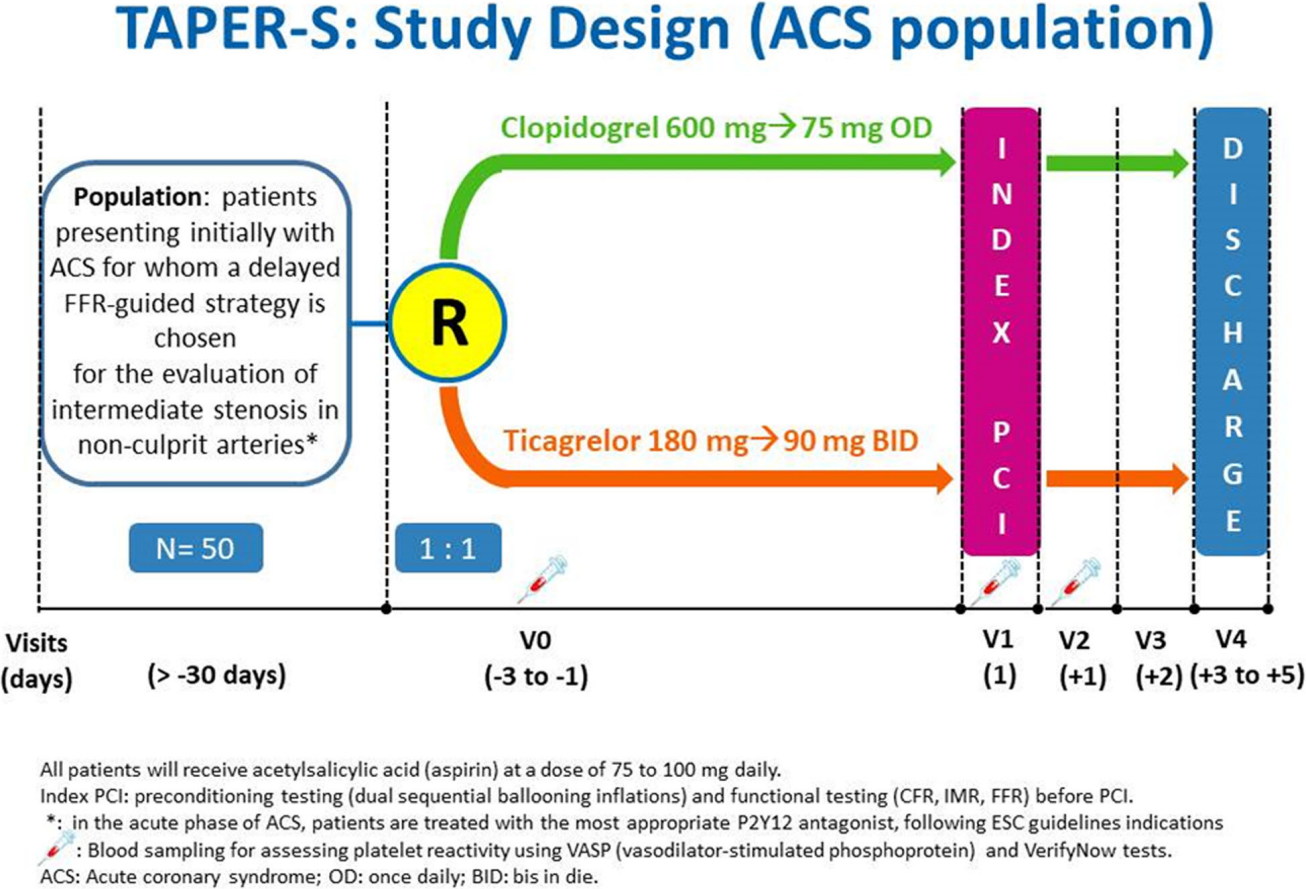
Flow-chart for patients presenting with ACS

**Fig. 2:**
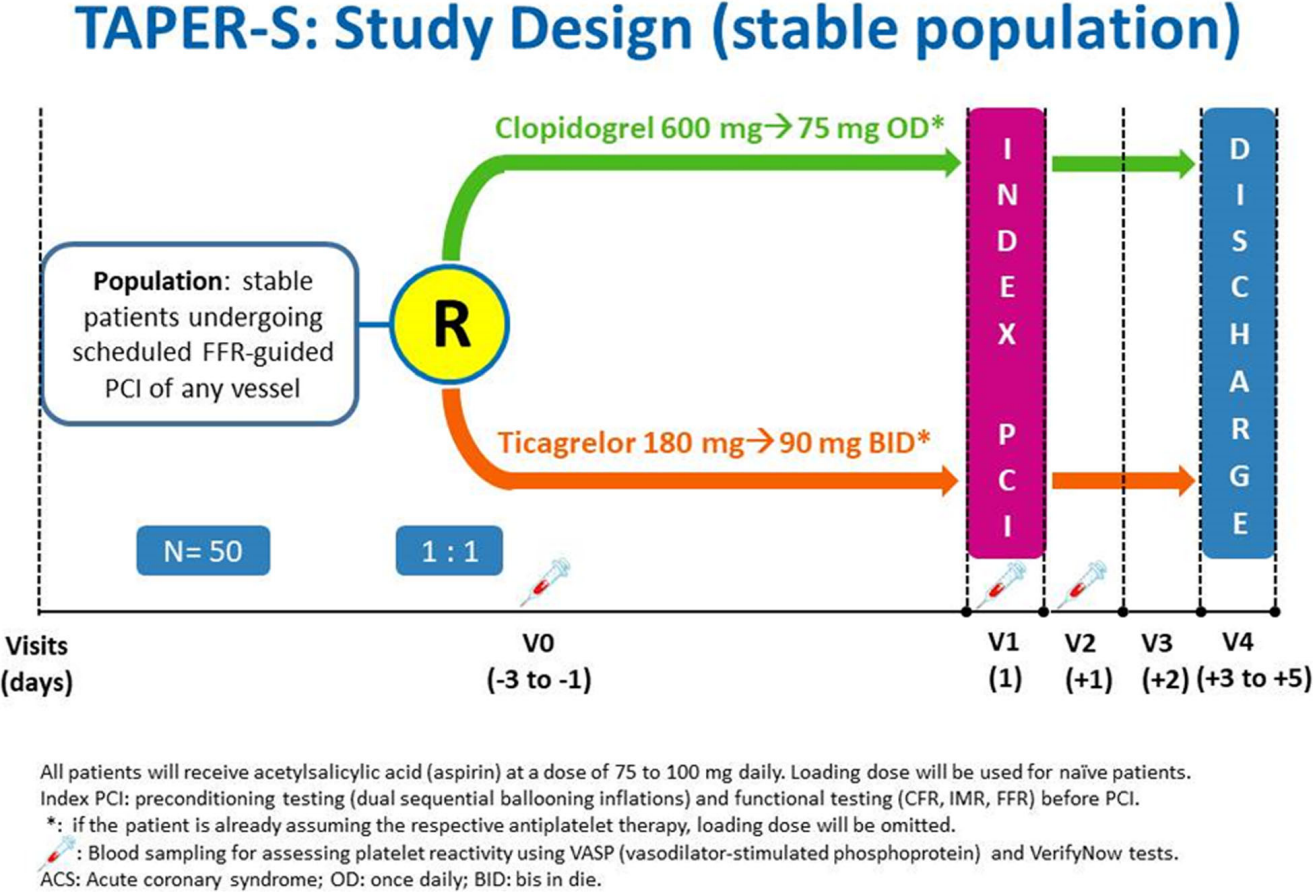
Flow-chart for patients presenting with stable coronary artery disease

This study is a prospective, randomized, blinded, end-point trial that will enroll patients with multivessel CAD requiring scheduled treatment of a second lesion located in a nonculprit vessel. The typical patient will be a patient with purely stable CAD and a scheduled, FFR-guided PCI of any vessel. Patient presenting initially with an ACS, with an intermediate coronary lesion (40–80% in severity, where functional evaluation of the stenosis with FFR must be used based on clinical situation) in a nonculprit coronary artery, for whom a delayed, FFR-guided strategy is chosen, may also be included in the study. In these patients, recovery of the microvascular function requires that at least 1 month has elapsed before re-evaluation of the culprit lesion, as FFR in the acute phase is known to be unreliable [31]. Screening/randomization will take place 1 to 3 days before the index procedure. Randomization will be blocked within each study site, which will allow an even balance of patients to be randomized to either drug at each recruiting center. Patients with FFR > 0.80 (no functionally significant stenosis) will be excluded from the study (Figs. 1 and 2). The study will be conducted in Italy, involving A. Gemelli Polyclinic of the Catholic University of the Sacred Heart in Rome and Grosseto Hospital (Ospedale della Misericordia Grosetto) in Grosetto, Italy.

**Fig. 3:**
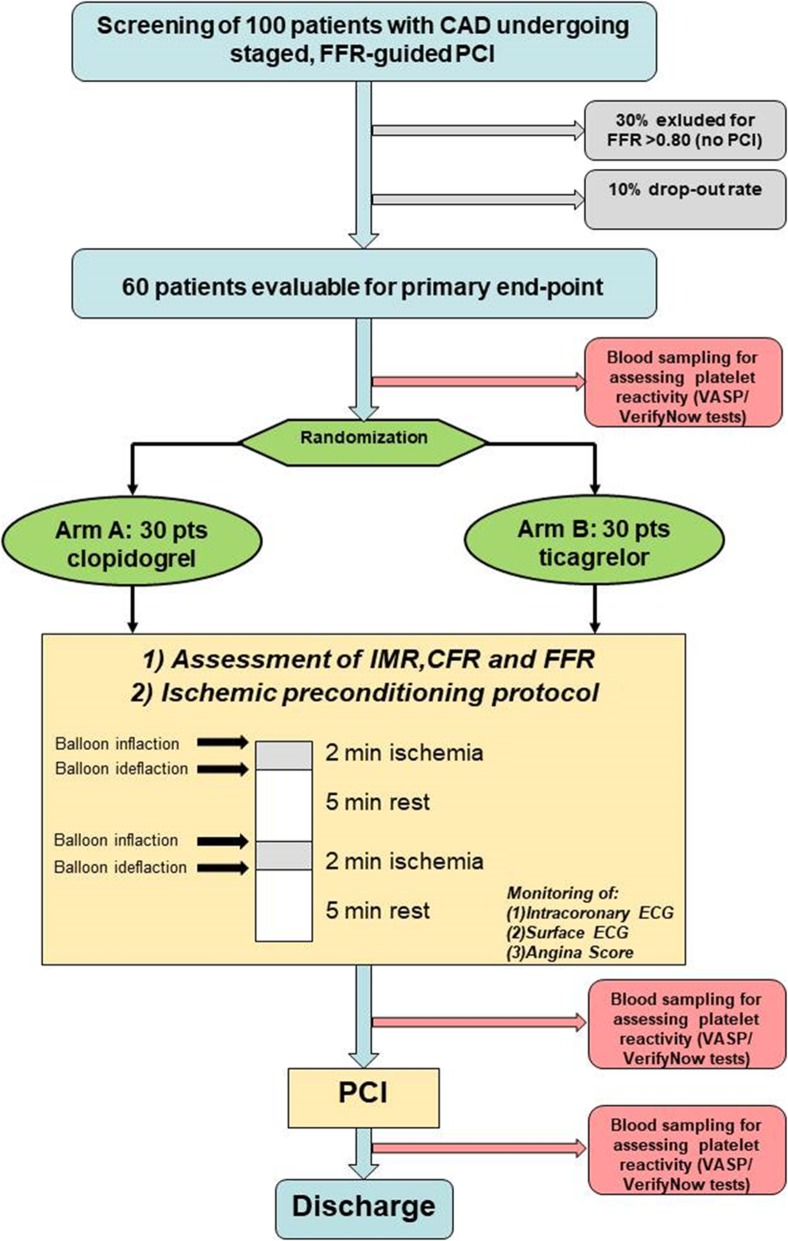
Taper-S study flow-chart

The study is designed to have a total duration of 23 months, with an enrollment period of approximately 20 months. Each patient will be treated with the study drug only during the in-hospital phase, for a minimum of 3 days (Day -1 to Day 1) up to a maximum of 7 days (Day -1 to Day 5). The patient will be hospitalized (as per usual clinical practice) from Day -3 to Day -1 (admission) to Day 0–5 of the study. The total number of enrolled patients will be one hundred (*N* = 100), with a projected 10% drop-out rate and 30% of negative FFR assessment (FFR > 0.80), with a final pool comprising 60 patients who will be evaluated in terms of primary and secondary endpoints. Therefore, the study enrollment will be stopped once one hundred patients are enrolled (Fig. 3).

### Sample size

This is an exploratory and pilot study, aimed at evaluating the off-target effects of ticagrelor on ischemic preconditioning. A sample size of 40–60 patients is, in general, considered appropriate for a pilot study and should provide important insights for the planning of a future larger study and for estimating confidence intervals [32]. Per our design, by assuming an absolute difference (delta) of 4 mm in the change of ST-segment shift from the first to the second balloon inflation between the two compared groups, a magnitude similar (albeit reversed) to the effect observed by Niccoli et al. in a study demonstrating the negative preconditioning effect of ethanol [33], we can infer that 30 patients per group will be required to provide an 80% power to detect a statistically significant difference between groups at a significance level set at *p* < 0.05. The standard deviation (SD) of the primary endpoint is expected to be approximately 5 to 4 mm in each treatment group. By enrolling one hundred patients, we also take into account those patients that will not require PCI after FFR evaluation (as observed in the FAME II trial) [34].

The main statistical analysis to be undertaken is a comparison of the primary endpoint (continuous variables) between the two groups of 30 patients each is a 2 × 2 analysis of variance (ANOVA) stratified by treatment group. The co-primary safety endpoint, which is a frequency value, will be analyzed using a Fisher’s exact test.

### Study population

Patients will be required to have a clinical indication to undergo scheduled FFR-guided PCI. Two types of patients will be included:

1. Patients presenting initially with an ACS, with an intermediate coronary lesion (40–80% in severity, where functional evaluation with FFR must be used based on clinical situation) in a nonculprit artery, thus requiring a staged FFR-guided PCI (at least 1 month after the index ACS event)
2. Patients with a stable CAD and who are undergoing scheduled FFR-guided PCI of any vessel

### Patient selection

#### Inclusion criteria

The inclusion criteria are as follows:

1. Signed and dated written Informed Consent obtained prior to the inclusion in the trial
2. Age ≥ 18 and ≤ 75 years
3. Weight ≥ 60 kg
4. Patients must have a clinical indication to undergo scheduled FFR-guided PCI (the two types of patients to be included are noted in 5 and 6)
5. Patients presenting initially with an ACS, with an intermediate coronary lesion (40–80% in severity, where functional evaluation with FFR must be used based on clinical situation) in a nonculprit artery, thus requiring a staged FFR-guided PCI (at least 1 month after the index ACS event);
6. Patients with a stable CAD undergoing scheduled FFR-guided PCI of any vessel
7. Patients already may be on the antiplatelet-directed treatment with either ticagrelor (previous ACS patients) or clopidogrel (stable patients)

#### Exclusion criteria

The exclusion criteria are as follows:

1. Known hypersensitivity to aspirin, clopidogrel, ticagrelor, or any similar drug
2. Concomitant oral anticoagulant therapy, or need for it (e.g., atrial fibrillation, mechanical valve, etc.)
3. Need for a concomitant cardiac noncoronary procedure, such as valve repair or replacement
4. Any previous history of ischemic stroke, intracranial hemorrhage or disease (neoplasm, arteriovenous malformation, or aneurysm)
5. Any active pathological bleeding or history of significant gastrointestinal bleeding, genitourinary bleeding or other site abnormal bleeding within the previous 3 months, other bleeding diathesis, or considered by the Investigator to be at high risk for bleeding
6. Concomitant oral or intravenous (IV) therapy with strong CYP3A inhibitors, CYP3A substrates with narrow therapeutic indices, or strong CYP3A inducers. Any of such concomitant treatment will be mapped and evaluated by the Investigator to comply with the exclusion criteria
7. Increased risk of bradycardia events
8. Known pregnancy and/or breast-feeding
9. Severe uncontrolled chronic obstructive pulmonary disease
10. Concomitant theophylline/aminophylline use
11. Baseline ECG with significant conduction abnormalities (i.e., left ventricular hypertrophy with repolarization abnormalities, left bundle branch block, etc.)
12. Evidence of prior myocardial infarction by cardiac imaging in the territory of the index vessel
13. Reduced left ventricular systolic function at screening/randomization (left ventricular ejection fraction < 40% within 48 h before index PCI, as assessed by transthoracic echocardiography)
14. Clinical and laboratory signs of congestive heart failure
15. Presence of coronary collaterals on diagnostic coronary angiography
16. Diffuse obstructive disease (≥ 70% stenosis) in the distal segment of the target vessel
17. Left main and/or three-vessel CAD
18. End-stage renal disease (as assessed by the estimated glomerular filtration rate CKD-EPI formula, eGFR < 15 mL/min./1.73 m^2^)
19. Documented severe hepatic impairment (e.g., elevated liver enzymes, cirrhosis, etc.)

### Study drugs

#### Dosing, posology, and route of administration

Ticagrelor will be given in a loading dose of 180 mg (on Day -3 to -1), followed by a dose of 90 mg twice daily (from Day 0 to discharge between Days 1 to Day 5, during the index hospitalization). If the patient is chronically on ticagrelor, the loading dose will be omitted. In case of missed dose, the lapses in therapy should also be avoided. A patient who misses a dose of ticagrelor should take only one tablet (their next dose) at its scheduled time.

Clopidogrel will be given in a loading dose of 600 mg (on Days -3 to -1), followed by a dose of 75 mg once a day (from Day 0 to discharge during Days 1 to Day 5, during the index hospitalization). If the patient is chronically on clopidogrel, the loading dose will be omitted. The antiplatelet switch will be managed according to generally agreed consensus. The details of the Antiplatelet Drug “switch” are specified in the section below.

#### Antiplatelet drug switch

Patients could be randomized while on treatment to either ticagrelor (previous ACS patients) or clopidogrel (stable patients). For those on long-term ticagrelor or clopidogrel treatment randomized to the corresponding drug, no loading dose will be given, and the patient will be continued on the respective drug. For those randomized to the noncorresponding group, the most recent recommendations regarding antiplatelet drug “switch” will be followed [35, 36] - a loading dose of the other drug will be given, 12–24 h before the scheduled PCI procedure. For patients previously on a long-term ticagrelor treatment, a 24-h washout period (i.e., the withdrawing of a single dose) will be applied before clopidogrel loading dose is initiated, to aid in clearance of the reversible platelet inhibition by ticagrelor and its main metabolite [36]. For both ticagrelor and clopidogrel, the last drug dose will be given at least 4 ± 1 h before the start of the index procedure [37]. After the index procedure, long-term antiplatelet treatment will be administered according to the most recent guidelines [38]. Timing and schedule of all the procedures projected during the protocol visits are detailed in the flow chart table that is available below. See also Additional file 1 for details related to main pharmacological interactions and adverse events definition and reporting.

### Compliance

Given that the administration of the drugs is conducted while the patients are hospitalized, the compliance to the treatment scheduled must be considered by default as 100% endorsed. Nevertheless, in case any of the requested doses are not to be administered, the issue and the relevant reason will be documented by the Investigator (and/or his delegates) on the appropriate source document.

### Discontinuation/stopping of the treatment

Enrolled subjects may be discontinued from the investigational product (IP) in case of consent withdrawal, any adverse event, and non-compliance to study protocol or incorrect enrollment.

### Data collection and management

The referent and person responsible for the coordinating center, Fondazione Policlinico Universitario Agostino GemelIi, will be Italo Porto, MD, PhD. The source data, recorded in the appropriate source documentation, will be reported by the Principal Investigator and/or his delegates in a web-based database (electronic case report form, eCRF). The validation of the inconsistencies (change or acceptance) will be made by the Principal Investigator and/or his delegates. Before the data freezing, the person in charge of the data management will code the medical terms. Data will be collected by their registered delegates and will be stored in dedicated paper file, which will be locked for security. The electronic data (the eCRF) will be password secured.

### Inspections

The Principal Investigator and/or his delegates must allow the regulatory authorities to conduct inspections. The inspection on the part of the Regulatory Authority consists of an official review of the documents, facilities, records, and any other resource considered by the authority to relate to the study. Auditing of the trial conduct will be performed according to the CTC Quality System and will be independent from investigators and the sponsor.

## Study procedures

### Visit scheduling

Patients satisfying inclusion criteria and having none of the exclusion criteria will be randomized to either ticagrelor or clopidogrel (Figs. 1 and 2). Ticagrelor will be administered in a loading dose of 180 mg followed by a dose of 90 mg twice daily. Clopidogrel will be administered in a loading dose of 600 mg, followed by a dose of 75 mg once a day. If the patient is chronically on ticagrelor or clopidogrel treatment, the loading dose will be omitted.

All patients will receive acetylsalicylic acid (aspirin) at a dose of 75 to 100 mg daily. For those who had not previously been receiving aspirin, 250 or 325 mg will be the preferred loading dose. The study drug will be continued for at least 72 h before the second PCI.

During PCI, two sequential balloon inflations will be performed to measure ischemic preconditioning. Operators will measure the degree of ST-segment elevation by intracoronary ECG and by surface ECG during coronary balloon inflation.

### Randomization

Patients will be randomly assigned to **ARM A (clopidogrel)** or **ARM B (ticagrelor)** in a 1:1 ratio. The allocation to the assigned treatment arm will be known to the patients and to the Principal Investigator (PI) and/or his delegates.

The randomization will be performed at Visit 0. Randomization process will be performed printing 50 tags reporting the word *“Ticagrelor”* and 50 tags reporting the word *“Clopidogrel”* inserted into anonymous white letter envelopes; the envelopes will be mixed and then stacked up in random order, with the opening side toward the bottom of the pile, by the study monitor; the resulting random sequence of 100 envelopes will be consigned to the PI or his delegates, which are entrusted with their preservation and responsible for any adulteration. At visit 0 the PI or his delegates will open the first available envelope, proceeding from top to bottom of the pile. The already opened envelopes will be stored separately from the others and will be available to the study monitor on request.

### Study timeline

The study timeline is depicted in Table 1. See also the checklist enclosed in Additional file 2.

**Table 1:** A TAPER-S study timeline

Screening and Randomization	
V0 – Days -3 to -1	
Signed and dated Informed Consent obtained prior to start any procedure scheduled for the study	
Verification and confirmation of the inclusion/exclusion criteria	
Randomization	
Demographics, medical history	
Physical Examination	
Vital Signs (BP, HR)	
NYHA Class definition	
Pregnancy Test (serum test or urine test as per decision of the clinician)	
Full Blood Count, Chemistry and Coagulation Tests	
ECG	
Echocardiography, if not performed within the last year from the Informed Consent signature	
Chest X-Ray	
VASP and VerifyNow Test	
Dyspnea Evaluation	
Drug Dispensing	
Drug Accountability	
Concomitant Medications	
Adverse Events	
Index PCI	
V1 – Day 0	
Verification and confirmation of the inclusion/exclusion criteria	
Vital Signs (BP, HR)	
ECG	
Ischemic Preconditioning	
Quantification of coronary microvascular resistance	
VASP and VerifyNow Test	
VAS Scale Evaluation	
Dyspnea Evaluation	
Drug Dispensing	
Drug Accountability	
Concomitant Medications	
Adverse Events	
Follow-up	
V2 – Day 1	
Physical Examination	
Vital Signs (BP, HR)	
ECG	
VASP and VerifyNow Test	
Dyspnea Evaluation	
Drug Dispensing	
Drug Accountability	
Concomitant Medications	
Adverse Events	
Pre-discharge Visit	
V3 – Day 2	
Physical Examination	
Vital Signs (BP, HR)	
Dyspnea Evaluation	
Drug Dispensing	
Drug Accountability	
Concomitant Medications	
Adverse Events	
Discharge/End of Study Visit	
V4 – Day 3 to Day 5	
Physical Examination	
Vital Signs (BP, HR)	
ECG	
Full Blood Count, Chemistry and Coagulation Tests	
Dyspnea Evaluation	
Drug Dispensing	
Drug Accountability	
Concomitant Medications	
Adverse Events	

### Assessments description

#### Dyspnea evaluation

The sensation of dyspnea will be reported by patients using the modified Borg scale; the scale is scored from 1 (no sensation of dyspnea) to 10 (maximum sensation of dyspnea) [39].

#### Assessment of platelet reactivity

We will perform platelet VASP (vasodilator-stimulated phosphoprotein) and VerifyNow test. The VASP test specifically assesses the inhibition of platelet reactivity index (PRI) linked to P_2_Y_12_ ADP receptor inhibition. The VASP test is a flow cytometry-based method that uses a phosphorylation-specific antibody and measures the PRI, expressed as a percentage and defined as the difference in VASP fluorescence intensity between resting (+PGE1, prostaglandin E1) and activated (+ADP) platelets. VASP phosphorylation analysis can evaluate the individual response to the clopidogrel loading dose prior to PCI and predict post-procedural major adverse cardiac events (MACE). In a study of Bonello et al., the optimal cut-off value of PRI assessed by VASP to exclude adverse events was 50% [40, 41].

VerifyNow P_2_Y_12_ test (Accumetrics, Inc., San Diego, CA) measures the agglutination of fibrinogen-coated beads by platelets stimulated by an agonist (ADP with prostaglandin E1) in citrated whole blood. The relevant thresholds are not clearly defined. Each assay records two results: P_2_Y_12_ reaction units (PRU) and percentage of inhibition. A good correlation has been established between the VerifyNow P_2_Y_12_ test and several other methods including the VASP assay [42].

Two blood samples for PRI and PRU testing will be drawn in Vacutainer tubes containing 3.8% trisodium citrate. The first sample will be collected at baseline following the randomization and then just before the index procedure. The analysis and the interpretation will be carried out in the Department of Cardiovascular Medicine of A. Gemelli Polyclinic or in the Grosseto Hospital according to the site of enrollment. The authors will store any residual blood sample for future research purposes.

For evaluation of the adenosine-induced effect of ticagrelor compared to clopidogrel, left-overs from the blood sampling will be utilized to measure the adenosine plasma concentration in the two groups together with ENT-1 expression and activity.

#### Ischemic preconditioning

During PCI, two sequential balloon inflations will be performed to measure preconditioning. Operators will measure the degree of ST-segment elevation by intracoronary ECG and by surface ECG during coronary balloon inflation.

Specifically, after passage of the guidewire through a target stenosis and then advancement of the balloon catheter across the stenosis, the intracoronary unipolar ECG from myocardium distal to the stenosis to be dilated will be obtained, connecting the proximal end of the guidewire as it exited from the balloon catheter. The unipolar intracoronary ECG will be filtered between 0.1 to 100 or 0.1 to 500 Hz and then displayed simultaneously with the standard surface leads being monitored. Surface and intracoronary ECGs will be recorded simultaneously and continuously during balloon inflation and deflation [43–45].

Moreover, angina score during coronary balloon inflation will be registered by using a Visual Analog Scale (VAS) on a scale of 0 (no pain) to 10 (most severe pain) [46]. For pharmacodynamic considerations, the Index PCI will be performed at 4 ± 1 h after ticagrelor or clopidogrel last dose.

#### Quantification of coronary microvascular resistance

CFR and IMR will be measured with an intracoronary pressure/temperature sensor-tipped guidewire (Radi pressure wire 4, Radi Medical Systems, Wilmington, Mass., USA), with an accuracy of 0.05 °C within a temperature range of 15–42 °C, to derive thermodilution curves. Previous experimental and human studies have demonstrated that the mean transit time (Tmn) of an intracoronary injection of saline at room temperature, derived from thermodilution curves, is inversely proportional to coronary flow [47–49]. Therefore, a given percent decrease in Tmn closely reflects a proportional percent increase in coronary blood flow. Three injections (3 mL each) of saline at room temperature will be performed, and the resting Tmn will be measured. An intravenous infusion of adenosine (Adenoscan, Sanofi Aventis S.p.A, Milan, Italy) at 140 μg/kg/min will be administered to induce a steady-state maximal hyperemia, followed by three further injections (3 mL) of room temperature saline to measure hyperemic Tmn.

Simultaneous measurements of mean aortic pressure (Pa, through the guiding catheter) and mean distal coronary pressure (Pd, by the pressure wire) will also be obtained in the resting and maximal hyperemic states. The following parameters will be calculated:

1. CFR will be calculated as the resting Tmn divided by the hyperemic Tmn [47, 48]
2. IMR will be calculated as the distal coronary pressure at maximal hyperemia divided by the inverse of the hyperemic Tmn [50]
3. BR (Baseline resistance index) will be calculated using the equation: BR = Pabase ×Tmnbase (Pdbase –Pw)/(Pabase –Pw) [51]
4. RRR (resistive reserve ratio) will be calculated using the following equation: RRR = BR index / IMR [49, 50]

## Statistical analysis

All the variables will be descriptively analyzed by arm and visit (mean, median, standard deviation, minimum and maximum for continuous variables, and frequency distribution for categorical variables). Categorical variables referring to a physical examination, vital signs and laboratory abnormalities will also be analyzed with shift tables (baseline vs. final visit). All analyses will be carried out in details in the respective Statistical Analysis Plan (SAP), which will be finalized in Version 1.0 before the database freezing.

Version 2.0 of the SAP will be prepared after the Data Review Meeting (in which the protocol violations will be evaluated) and will include the list of the patients belonging to the defined populations and the randomization list.

A descriptive analysis of demographic data and baseline characteristics will be carried out to verify the balance of the two arms at the study entry.

The delta ST-segment elevation by intracoronary ECG during two-step sequential coronary balloon inflation in the culprit vessel will be compared between the ticagrelor and clopidogrel arms by using two-way ANOVA (2 × 2). Missing data in the primary analysis will be handled by simple imputations. An interim efficacy analyses will be conducted after the accrual of two-third of therandomized patients have been reached.

## Supplementary information

**Additional file 1.** Details related to main pharmacological interactions and adverse events definition and reporting are provided in the additional file, enclosed in the manuscript.

**Additional file 2.** SPIRIT 2013 Checklist.

## Data Availability

The datasets used and/or analyzed during the current study are available from the corresponding author on reasonable request. Case report forms and study documents will be electronically implemented and filled in at any visit. Data will be protected by passwords assigned to the PI and his registered delegates. The Institution Fondazione Policlinico Universitario A. Gemelli, being the Sponsor of the presented study, and represented by Dr. Italo Porto as a Principal Investigator (as per contract stipulated with AstraZeneca and IP) has the full ownership of the results and all accompanying data. Study investigators and collaborators are committed to publish obtained results publicly in a peer-reviewed biomedical journal. The final trial dataset will only be available to principal site investigators and will not be shared/disclosed to any third party or auxiliary trial sponsors (such as AstraZeneca that provided partial grant for this study). The full study protocol will be published and made available on ClinicalTrials.gov (https://clinicaltrials.gov). All personal data will be collected, stored, shared, and maintained according to EU Regulations and local laws.

## Abbreviations

ACS: Acute coronary syndrome
AE: Adverse events
AIFA: Agenzia Italiana del Farmaco
ANOVA: Analysis of variance
BP: Blood pressure
BR: Baseline resistance index
CAD: Coronary artery disease
CBFV: Coronary blood flow velocity
CFR: Coronary flow reserve
CFVR: Coronary flow reserve velocity
CTC: Clinical Trial Center S.p.A.
DAPT: Dual antiplatelet therapy
EC: Ethics committee
eCRF: Electronic case report form
EMA: European Medicines Agency
FFR: Fractional flow reserve
FPG: Fondazione Policlinico Universitario A. Gemelli
HR: Heart rate
IC: Informed consent
ICH: International Conference on Harmonization
IMR: Index of microvascular resistance
MACE: Major adverse cardiac events
NSTEMI: Non-ST-elevation Acute Coronary Syndromes
Pa: Aortic pressure
PCI: Percutaneous coronary intervention
Pd: Distal coronary pressure
PRI: Platelet reactivity index
PRU: P2Y_12_ reaction units
*Pw*: Wedge pressure
RISK: Reperfusion injury salvage kinases
RRR: Resistive reserve ratio
SAE: Serious adverse event
SD: Standard deviation
SUSAR: Suspected unexpected serious adverse reactions
T_mn_: Transit time
VASP: Vasodilator-stimulated phosphoprotein
