# Supplementary material for: Ticagrelor and preconditioning in patients with stable coronary artery disease (TAPER-S): a randomized pilot clinical trial
Source: Trials. 2020 Feb 17;21:192. doi: 10.1186/s13063-020-4116-7 (PMC7027127; doi:10.1186/s13063-020-4116-7)
Supplement: Supplementary file 1 — Additional file 1. Details related to main pharmacological interactions and adverse events definition and reporting are provided in the additional file, enclosed in the manuscript. [file 13063_2020_4116_MOESM1_ESM.docx]

**Additional File 1**

**CONCOMITANT PHARMACOLOGICAL THERAPIES**

***CYP3A4 inhibitors***

Strong CYP3A4 inhibitors – co-administration of ketoconazole with ticagrelor increased the ticagrelor peak serum concentration (Cmax) and area under the curve (AUC) equal to 2.4-fold and 7.3-fold, respectively. The C_max_ and AUC of the active metabolite were reduced by 89% and 56%, respectively. Other strong inhibitors of CYP3A4 (clarithromycin, nefazodone, ritonavir, and atazanavir) would be expected to have similar effects and therefore concomitant use of strong CYP3A4 inhibitors with ticagrelor is contraindicated.

***CYP3A inducers***

Co-administration of rifampicin with ticagrelor decreased ticagrelor Cmax and AUC by 73% and 86%, respectively. The C_max_ of the active metabolite was unchanged and the AUC was decreased by 46%, respectively. Other CYP3A inducers (e.g. phenytoin, carbamazepine, and phenobarbital) would be expected to decrease the exposure to ticagrelor as well. Co-administration of ticagrelor with potent CYP3A inducers may decrease exposure and efficacy of ticagrelor, therefore, their concomitant use with ticagrelor is discouraged.

***Cyclosporine (P-gp and CYP3A inhibitor)***

Co-administration of cyclosporine (600 mg) with ticagrelor increased ticagrelor C_max_ and AUC equal to 2.3-fold and 2.8-fold, respectively. The AUC of the active metabolite was increased by 32% and C_max_ was decreased by 15% in the presence of cyclosporine. No data are available on concomitant use of ticagrelor with other active substances that also are potent P-glicoprotein (P-gp) inhibitors and moderate CYP3A4 inhibitors (e.g. verapamil, quinidine) that also may increase ticagrelor exposure. If the association cannot be avoided, their concomitant use should be made with caution. Medicinal products that can affect haemostasis should be used with caution in combination with ticagrelor or discarded, if possible. The concomitant use of ticagrelor with doses of simvastatin or lovastatin greater than 40 mg is not recommended [1]. Due to reports of cutaneous bleeding abnormalities with selective serotonin reuptake inhibitors (SSRIs) (e.g. paroxetine, sertraline, and citalopram), caution is advised when administering SSRIs with ticagrelor as this may increase the risk of bleeding [2].

**SAFETY AND TOLERABILITY EVALUATION**

***Safety and Tolerability Parameters***

All adverse events will be recorded starting from the Informed Consent Signature. The occurrence of the Adverse Events related to the procedure will be appropriately recorded and documented to define the safety profile of the patients.

***Adverse Events***

The Investigator is responsible for the managing of the events meeting the definition and criteria of an adverse event (AE) or a serious adverse event (SAE), as provided in this protocol. All adverse events occurring between the date of informed consent signature will be recorded in the CRF (and in the SAE form, if applicable) between the date of informed consent signature and the date of study completion. Each subject will be monitored regularly by the Investigator and study personnel for adverse events occurring throughout the study.

***Definitions***

- *Adverse Event (AE)*

Any untoward medical occurrence in a subject or clinical trial subject administered a medicinal product and which does not necessarily have to have a causal relationship with this treatment. An adverse event can, therefore, be any unfavourable and unintended sign (e.g. an abnormal laboratory finding), symptom, or disease temporally associated with the use of a medicinal product, whether considered related to the medicinal product or not.

- *Adverse Reaction*

All untoward and unintended responses to an investigational medicinal product related to any dose administered are considered adverse reactions. AEs must be classified by the Investigator as "Serious" or "Non-Serious", according to the following definitions:

- *Serious Adverse Event (SAE)*

Serious adverse event/reaction means an adverse event/reaction which:

• results in death,

• is life-threatening,

• requires in-patient hospitalization or prolongation of existing hospitalization,

• results in persistent or significant disability or incapacity,

• is a congenital anomaly/birth defect

• important medical event

- *Non-Serious Adverse Event (NSAE)*

Defined as the event that does not meet any of the criteria defining SAEs.

- *Causality assignment*

The definition of “Suspected Adverse Reaction” implies the existence of a reasonable possibility of a correlation between the event observed and the medicinal product tested. This means that it is possible to identify evidence (data) which would support the existence of such correlation.

- *Unexpected Adverse Reaction*

An unexpected adverse reaction is an adverse reaction, the nature or severity of which is not consistent with the applicable Product Information (e.g. Investigator’s Brochure for an unauthorized investigational product or the Summary of Product Characteristics for an authorized product).

***Monitoring of Adverse Events***

Subjects will be monitored throughout the study for adverse events to the study treatment and/or procedures. AEs will be documented and collected on an ongoing basis during the treatment period and the designated follow-up period. The principal Investigator and/or the designees will instruct the subject on how to communicate any AEs occurred. Medical events fulfilling one or more criteria of significance and occurring after the informed consent has been signed must be immediately (within maximum 24 hours) reported to the Sponsor (or delegate) using a pre-specified SAE form.

SAEs still present at the end of the study period will be followed until the final outcome is determined. Any subject who experiences an adverse event (AE) (whether serious or non-serious) or has a clinically significant abnormal laboratory test value(s) will be evaluated by the Investigator and will be treated and/or monitored, even after the study ends, until the symptoms or value(s) return to normal, acceptable levels or chronicity, as judged by the Investigator.

***Serious Adverse Event Reporting***

Once the Principal Investigator (and/or designees) becomes aware that a SAE has occurred in a study subject, he/she will immediately notify the Sponsor by contacting the Clinical Trial Center S.p.A., using a SAE form.

The applicable follow-up information should be managed with the same modality and timeframe applied for the initial notification (a new SAE form should be completed and transmitted within 24 hours from the first information received about the event).

***Regulatory Obligations***

All the “Suspected Unexpected Serious Adverse Reactions” (SUSAR) will be notified by the Sponsor (or delegate) to the European Regulatory Authority (EMA, European Medicine Agency), to the coordinator of the local Ethics Committee and to the Principal Investigators involved in the study, following the timeframes and modality defined by the applicable regulations.

Furthermore, the Sponsor has the obligation to submit (directly or through a delegate) to AIFA (Agenzia Italiana del Farmaco) and to all the applicable Ethics Committees a periodic update on the experimental drug (“Development Safety Update Report”, DSUR), once a year throughout the duration of the study (i.e.: from the date of Regulatory approval up to the date of Last Visit/Last Patient)

**REFERENCES**

1. DiNicolantonio JJ, Serebruany VL. Exploring the Ticagrelor-Statin Interplay in the PLATO Trial. Cardiology. 2013;124(2):105-7.
2. Thornton JD, Agarwal P, Sambamoorthi U. Use of selective-serotonin reuptake inhibitors and platelet aggregation inhibitors among individuals with co-occurring atherosclerotic cardiovascular disease and depression or anxiety. SAGE open medicine. 2016;4:2050312116682255-.
